# Impact of near infrared light in pediatric blood drawing Centre on rate of first attempt success and time of procedure

**DOI:** 10.1186/s13052-018-0501-1

**Published:** 2018-05-25

**Authors:** Ester Conversano, Giorgio Cozzi, Matteo Pavan, Marta Minute, Elena Gortan, Marcella Montico, Liza Vecchi Brumatti, Luca Ronfani, Egidio Barbi

**Affiliations:** 1 University of the Study of Trieste, Trieste, Italy; 2 Institution for Maternal and Child Health – IRCCS “Burlo Garofolo”, Trieste, Italy

**Keywords:** Phlebotomy, Venipuncture, Blood sample, Near-infrared light, Pediatric

## Abstract

**Background:**

Peripheral blood access and venipuncture are a stressful and painful experience in pediatric patients; moreover, it is estimated that more than one attempt is required to achieve the procedure in about one third of children. For this reason, we investigated if Near-infrared light technology routinely used, could give an advantage to venipuncture in a pediatric blood center setting.

**Methods:**

We conducted an open, pseudo-randomized controlled trial with two parallel arms, in the blood-drawing center, with enrolment of 115 patients between 0 and 18 years, in 14 consecutive working days. Fifty-three subjects were enrolled in group 1 (VeinViewer®) and 62 in group 2 (control group). We divided patients into three subgroups considering their age (< 5 years, 6–10 years, > 10 years). The primary study outcome was to assess if the use of VeinViewer® was associated with a reduction of time to perform blood sampling. The secondary outcome was to analyze VienViewer®‘s impact on first attempt success rate in blood sampling.

**Results:**

No difference was found regarding the duration of blood sampling between the two groups, even after stratifying the patients into the three age subgroups. There was no difference between the two groups in the success at the first attempt in blood sampling.

**Conclusions:**

Routine use of VeinViewer® is not useful to reduce time of the procedure during venipuncture.

**Trial registration:**

The study was registered with ClinicalTrials.gov, with number NCT03277092, on September 8, 2017.

## Background

Peripheral venous cannulation and blood samples are frequently required in the pediatric hospital environment. Obtaining these in infants and toddlers can be challenging even for trained nurses and pediatricians. Some conditions might exacerbate venipuncture’s difficulties, such as increased subcutaneous tissues in newborn, poor venous asset in patient requiring frequent venous cannulation because of chronic disease, dark skin color, malnourishment, and obesity. Failure of intravenous (IV) placement can be predicted by a specific score, known as the difficult intravenous access (DIVA) score, which consider the visual and tactile assessment of the vein, the color of the skin, and the age of the patient [1]. Considering all these factors, it is not surprising that more than one attempt is required to achieve peripheral venous cannulation in about one third of children [2]. Moreover, up to 60% of children report pain and distress during venipuncture. Performing IV placement with as few attempts as possible is advocated, since it is well known that more the puncture attempts are, more the physical and emotional children distress is, which can negatively affect eventual subsequent procedures [3].

In the last years, specific tools have been developed to enhance the success rate in venipuncture. One of these is based on the near-infrared (NIR) technology: near-infrared light is projected by a machinery on to the skin and then absorbed by blood, but reflected by surrounding tissue. The machinery captures that information, processes it through a computer and then project a digital real-time image of the patient’s vein pattern directly on the skin. In the study of Cuper and colleagues, a trend in reduction of time spent to obtain a blood sample in children younger than 6 year-old using NIR-based device was observed [4].

Based on these results, we investigated if the adoption of one of these devices (VeinViewer® Flex, Christie Medical Corporation, Memphis, Tennessee, USA) in a pediatric blood-drawing center could decrease time spent to obtain a blood sample and improve the rate of first attempt success in children aged 0 to 18 years.

## Methods

We conducted an open, pseudo-randomized controlled trial with two parallel arms, in the blood-drawing center of a tertiary pediatric hospital in the North-East of Italy. The study was performed between October and December 2017, with the subjects enrolment performed in 14 consecutive working days. The eligible subjects were pediatric patients from 0 to 18 years of age who came to the blood-drawing center to collect blood samples by venipuncture. Most of the subjects were referred to the center by general practitioners for routine non-urgent blood tests. Children who had previously been applied a topical anesthetic cream on a suitable site for venipuncture were excluded, since it could have limited nurses’ choice regarding the site of IV placement.

Patients admitted in the first 7 days were allocated to the VeinViewer® group (group 1), and those admitted in the following 7 days were assigned to the control group (group 2) in which a traditional sampling method was used. No formal randomization was performed mainly due to the quickness needed in a high turnover blood-drawing center, since using the same procedure in every subsequent patient allowed the operator not to lose time passing from one technique to another.

For all the subjects, standard care were adopted consisting in blood sampling performed with patients seated or held on parents’ laps according to age, and routine application of appropriated distraction techniques. Four pediatric nurses with variable years of work experience were involved in the study and randomly assigned to IV placement. First, the patient was placed in a comfortable position, than VeinViewer® was switched on, and the material for blood sample was prepared; additionally, alcohol was applied as a disinfectant and to increase the contrast between the skin and the vein. The VeinViewer® was positioned approximately 30 cm above the puncture site, held by one nurse. Time monitoring started when the tourniquet was placed or when the second nurse started to look for the suitable vein for the procedure, whichever was first, until the blood was flowing in to the needle. Attempts were defined as each needle penetration into the skin, while redirection underneath the skin was not counted as a separate action.

Data were recorded in a standardized form by the nurse who performed the venipuncture. The variables collected for each patient were children’s age and sex, the site of the procedure, and the number of procedural attempts. We divided patients into three subgroups considering their age: < 5 years, 5–10 years, > 10 years.

The primary study outcome was to assess if the use of VeinViewer® is associated with a reduction of time to perform blood sampling. The secondary outcome was to analyze the VienViewer®‘s impact on first attempt success rate in blood sampling. The study protocol received approval from the Independent Bioethic Committee. All parents of the subjects enrolled provided written, informed consent for participation. The study was registered with ClinicalTrials.gov (NCT03277092). Collected data were analyzed by an independent epidemiologist at the Clinical Epidemiology and Public Health Research Unit of our Institute.

### Statistical analysis

Time in seconds and age in years are presented as medians and Inter-Quartile Range (IQR). Categorical data are presented as absolute frequencies and percentages. As time was left skewed and not normally distributed, the differences in times between groups were analyzed with Kruskall-Wallis test. Stratified and multivariate analysis were used to access if the time needed for blood sampling was different in the two groups independently from age and nurse experience. For multivariate analysis, a generalized linear model with gamma family and log link function was chosen to take into account for the left-skew. A *p*-value < 0.05 was considered as statistically significant. All the analyses were carried out with Stata 14.

## Results

### Patient characteristics

We enrolled 115 patients between 0 and 18 years. 53 subjects were enrolled in group 1 (VeinViewer®) and 62 in group 2 (control group). The age of children was similar in the two groups, with a median of 9 (IQR 5–12) in the VeinViewer® group and a median of 8 (IQR 4–12) in the control group (Table 1). Of the total sample, 57 patients were male (49.6%) and 58 were female (50.4%) without significant differences between groups. In 96.5% (*n* = 111) of the cases the venipuncture was performed on the antecubital fossa, in 0.9% (n = 1) on the forearm, and in 2.6% on the dorsal aspect of the hand (*n* = 3). An imbalance in groups was observed according to nurse experience, as nurses with more than 5 years of experience performed only 9 (14.5%) procedures in the VeinViewer® group and 26 (49.1%) in the control group.

**Table 1:** Characteristics of the participants: group 1 (VeinViewer®), group 2 (control)

	Group 1	Group 2
	n (%)	n (%)
Male (n, %)	34 (54.8%)	23 (43.4%)
Age (median, IQR)	9 (5–12)	8 (4–12)
Age classes (n,%)		
0–5	18 (29.0%)	19 (35.8%)
6–10	19 (30.7%)	13 (24.5%)
> 10	25 (40.3%)	21 (39.6%)
Nurse experience, > 5 years	9 (14.5)	26 (49.1)

### Primary outcome

No difference was found regarding the duration of blood sampling between the two groups. In the VeinViewer® group the median time of procedure was 44.1 s (IQR 29.3–58.3), compared to 45.8 s (IQR 36.8–56.3) of the control group. No significant difference was found even after stratifying the patients for the age groups.

As data were unbalanced for nurse experience, a multivariate analysis was performed to control for this factor. The results do not change when controlling for nurse experience and age of patients. Duration of sampling was comparable even between the two groups of nurses with a median of 45.4 s (IQR 34.8–54.7) in those with a professional experience less than five years and of 45.6 s (IQR 26.5–69) in the most experienced group (Table 2).

**Table 2:** Seconds needed for the procedure in the two study groups

	Group 1	Group 2	*p*-value
	median (IQR)	median (IQR)	
Total	44.1 (29.3–58.3)	45.8 (36.8–56.3)	0.357
Age classes			
0–5	56.4 (34.7–83.5)	53.4 (45.6–67.3)	0.648
6–10	44.5 (28.5–54.3)	43.5 (35–46.3)	0.908
> 10	34.7 (24.9–50.1)	43.3 (35.7–60.4)	0.054
Nurse experience			
0–5 years	45.7 (36.1–54)	41.6 (32.8–55.1)	0.430
> 5 years	56.3 (43.5–61)	44.2 (25.8–69.7)	0.473

### Secondary outcome

There was no difference between the two groups in the success rate at the first attempt in blood sampling. More than one attempt was necessary only in 3 cases, all belonging to the control group (Table 2).

## Discussion

To the best of our knowledge, this is the first study that assessed the usefulness of a routine use of VeinViewer® in a pediatric blood-drawing setting. The only study considering the use of NIR for venipuncture was performed by Cuper and colleagues in children younger than 6 years of age, which showed an absolute increase in first attempt success of 10%, and a tendency of a shorter time of needle manipulation [4].

Our results did not show a reduction in time spent to perform venipuncture using a NIR-based machinery in routine blood sampling. Furthermore, there was no impact on first attempt success rate, according to the meta-analysis performed by Park and colleagues [5]. In a fortuitous way, the group of nurses that used VeinViewer® were less experienced, but this imbalance did not affect the study outcome.

In our experience nurses reported that the use of VeinViewer® intrigued children curiosity, permitting to exploit the machinery also as a distracting activity, an element particularly relevant in pediatric settings.

Data on the efficacy of NIR in the pediatric population are still limited but it appear particularly useful in specific settings. The use of NIR seems to make it easier to place peripherally inserted central catheters (PICCs) in neonates [6]. NIR also increased first-attempt success rate in patients with a DIVA score greater than 4 (*p* = 0.026), even if the reduction in time procedure was not significant [7]. An advantage was confirmed by a particular application in hemophilic patient with difficult cannulation [8]. A study carried out in a pediatric Emergency Department showed no overall benefit in using NIR, but the subgroup analysis of children aged 0 to 2 years suggested that it decreased the time to peripheral intravenous catheter (PIV) placement in this age subgroup [9]. Lastly, in an oncologic Department, the use of NIR was associated to a significantly shorter time to get PIV, and to the perception by the patients that nurses who used NIR were more skilled [10].

This study had some limits. We did not use DIVA score, neither we considered stratification according to BMI (body mass index). Since children accessing to a blood-drawing center as outpatient are usually healthy subjects performing occasional routine blood sampling, we did not stratified them in healthy, chronically or acutely ill. Furthermore, we did not formally measured the impact of VeinViewer® on the perception of patients and parents.

## Conclusion

Blood sampling is a traumatic event for most children and sometimes a challenging procedure even for the most trained nurses, due to the anatomical peculiarities of the children or their excessive agitation. Whereas in pediatric settings are usually adopted distraction techniques (e.g. electronic tablets, smartphones, soap bubbles) to reduce the emotional distress, and procedural analgesia to prevent the pain, tools proven to enhance the success of venipuncture are scarce.

According to our results, the use of VeinViewer® does not facilitate phlebotomies, but not requiring significant additional time, nor affecting the first attempt success rate, both in the younger and the older pediatric patients, it could be used according to health worker’s preference.

In specific conditions, as in children younger than 2 years of age, in patients with a high DIVA score, and subjects with poor venous asset associated or not with chronic diseases, the use of VeinViewer® may be particularly helpful.

## Abbreviations

DIVA: Difficult intravenous access
IQR: Inter-Quartile Range
NIR: Near-infrared
